# Bidirectional Size Control for Angstrom-Scale Graphene Pores by Competitive Growth and Etching

**DOI:** 10.1021/acs.nanolett.6c01120

**Published:** 2026-05-08

**Authors:** Ceren Kocaman, Mojtaba Chevalier, Yueqing Shen, Luis Francisco Villalobos, Kumar Varoon Agrawal

**Affiliations:** [a] Laboratory of Advanced Separations (LAS), 27218 École Polytechnique Fédérale de Lausanne (EPFL), Rue de l’Industrie 17, 1950 Sion, Switzerland; [b] Chemical Engineering and Materials Science, 5116 USC Viterbi School of Engineering, 3650 McClintock Avenue, OHE 106, Los Angeles, California 90089, United States

**Keywords:** porous graphene, gas separation, vacancy defects, tuning pore size, bidirectional size control

## Abstract

Precise control over angstrom-scale pores in graphene remains a central challenge for exploiting its potential for gas separation. Most pore formation methods produce broad pore-size distributions with a long tail of nanometer-scale, nonselective pores. Here, we present a strategy based on simultaneous competitive growth and etching during chemical vapor deposition. By coupling CH_4_ as a carbon precursor with CO_2_ as a mild etchant, we establish a continuous kinetic regime in which pore expansion and shrinkage emerge from the same growth environment and are tuned bidirectionally by gas-phase composition. Carbon isotope labeling reveals that pore shrinkage proceeds via edge-mediated lattice reconstruction fueled exclusively by CH_4_, while CO_2_ acts solely as an etchant. This competitive growth–etching interplay enables the systematic contraction of nanometer-scale pores into angstrom-scale apertures. The resulting porous graphene exhibits remarkably enhanced molecular sieving behavior, providing a general framework for postsynthetic control of defect dimensions in two-dimensional materials.

## Graphical abstract

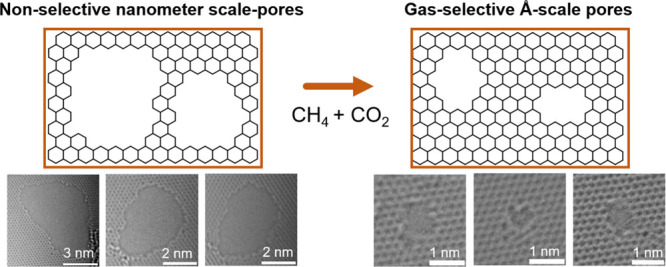

Single-layer graphene has captivated the scientific community with its exceptional mechanical, chemical, and thermal properties.^1,2^ Although the pristine graphene lattice is impermeable to gas molecules, introducing pores enables its application in molecular separation,^3–6^ electrochemical energy conversion and storage,^7–10^ and sensing.^11,12^ In particular, high mechanical strength^13,14^ and remarkable chemical^15^ and thermal^16^ stability, in addition to the atom thinness of graphene, make it promising as a selective layer in membrane-based separation. When angstrom-scale pores are incorporated into graphene, it becomes uniquely possible to achieve attractive gas permeance as well as gas pair selectivities.^17^

To unlock the full potential of porous graphene-based membranes for gas separation, controlling PSD at the angstrom scale is highly desirable.^18–20^ Various approaches have been developed to introduce angstrom-scale pores into the graphene lattice, including physical methods (irradiation of ion^3,21,22^ and electron^23–25^ beams), chemical methods (oxidative^26–31^ or plasma etching^32^), and direct bottom-up synthesis of porous graphene.^33–35^ While some methods, such as oxidative etching, offer good control over pore formation, tuning the pore size in both directions, i.e., increasing and decreasing, remains in infancy. Recent work has shown that sequential etching and selective pore enlargement can progressively refine PSD, enabling narrower distributions while retaining porosity.^36^ Such strategies, exemplified by cascaded compression of PSD,^36^ represent a promising direction for fabricating high-performance graphene membranes. However, the effectiveness of cascaded compression for the smallest set of angstrom-scale pores, i.e., those suitable for gas separation, has not been demonstrated.

An alternative approach to pore-size regulation is to place graphene growth and etching in direct kinetic competition, such that pore evolution is governed by the relative rates of carbon addition and removal at graphene edges. In this framework, pore expansion and contraction are not achieved through separate treatments but rather represent two limits of a single growth–etching continuum. Importantly, this approach enables bidirectional pore-size evolution, contraction or expansion, by simple modulation of the gas-phase chemical potential, without altering the temperature, substrate, or processing sequence. A kinetic Monte Carlo (kMC) simulation of graphene growth in the combined presence of an etchant and a growth precursor revealed an interplay between competitive growth and etching, predicting that a bidirectional kinetic growth regime can exist.^37^

A direct growth competition using CH_4_ (growth precursor) and CO_2_ (etchant) has previously been explored for controlling nanometer-scale pores in graphene membranes for ion–ion separation;^38^ however, it is not clear whether such an approach would be suitable for gas separation, given a fundamentally different pore-size range (3–4 Å for gas vs nanometer scale for ions) and transport regime. Ion–ion separation is insensitive to changes in pore size in graphene in the range of 2.5–4 Å. In contrast, gas separation by porous graphene occurs via subangstrom size differentiation, where transport rates and selectivity depend exponentially on small changes in pore size relative to the size of the gas molecules. Consequently, strategies effective at the nanometer scale do not directly translate to angstrom-scale graphene pores.

Here, we systematically investigate this growth–etching competition under conditions relevant to gas separation by varying CH_4_/CO_2_ partial pressures and growth durations to identify optimized regimes for controlled pore evolution. We demonstrate that initially large pores (>1 nm) can be controllably contracted to angstrom-scale apertures via competitive growth and etching in a CH_4_/CO_2_ atmosphere, where CH_4_ serves as the growth precursor and CO_2_ acts as a mild etchant.^39,40^ The net direction of pore evolution is governed by the gas composition, enabling either pore contraction through edge-mediated lattice reconstruction or pore expansion through etching within the same kinetic framework.

Using carbon isotope labeling combined with Raman spectroscopy, we show that graphene regrowth occurs exclusively from CH_4_ at the pore edges, providing direct evidence of edge-mediated lattice reconstruction as the mechanism for pore shrinkage. By carefully tuning the interplay between CH_4_ and CO_2_, we regulate the rate of pore evolution and ultimately convert nonselective pores to gas-selective pores. Furthermore, we demonstrate that this approach can be applied as a postsynthetic strategy to enhance the performance of already gas-selective graphene membranes.

We first synthesized completely intergrown polycrystalline single-layer graphene on Cu foil by exposing Cu to CH_4_ for 30 min at 1000 °C by chemical vapor deposition (CVD). We refer to this as completely intergrown graphene (CIG) (Figure S1). We also synthesized samples in which graphene was not completely intergrown (NCIG), which hosted isolated graphene grains with an average grain size of 16.5 ± 7.5 μm (Figure S2 and Figure 1b). This was achieved by exposing Cu foil to CH_4_ for 10 s at 1000 °C in the absence of CO_2_.

**Figure 1:**
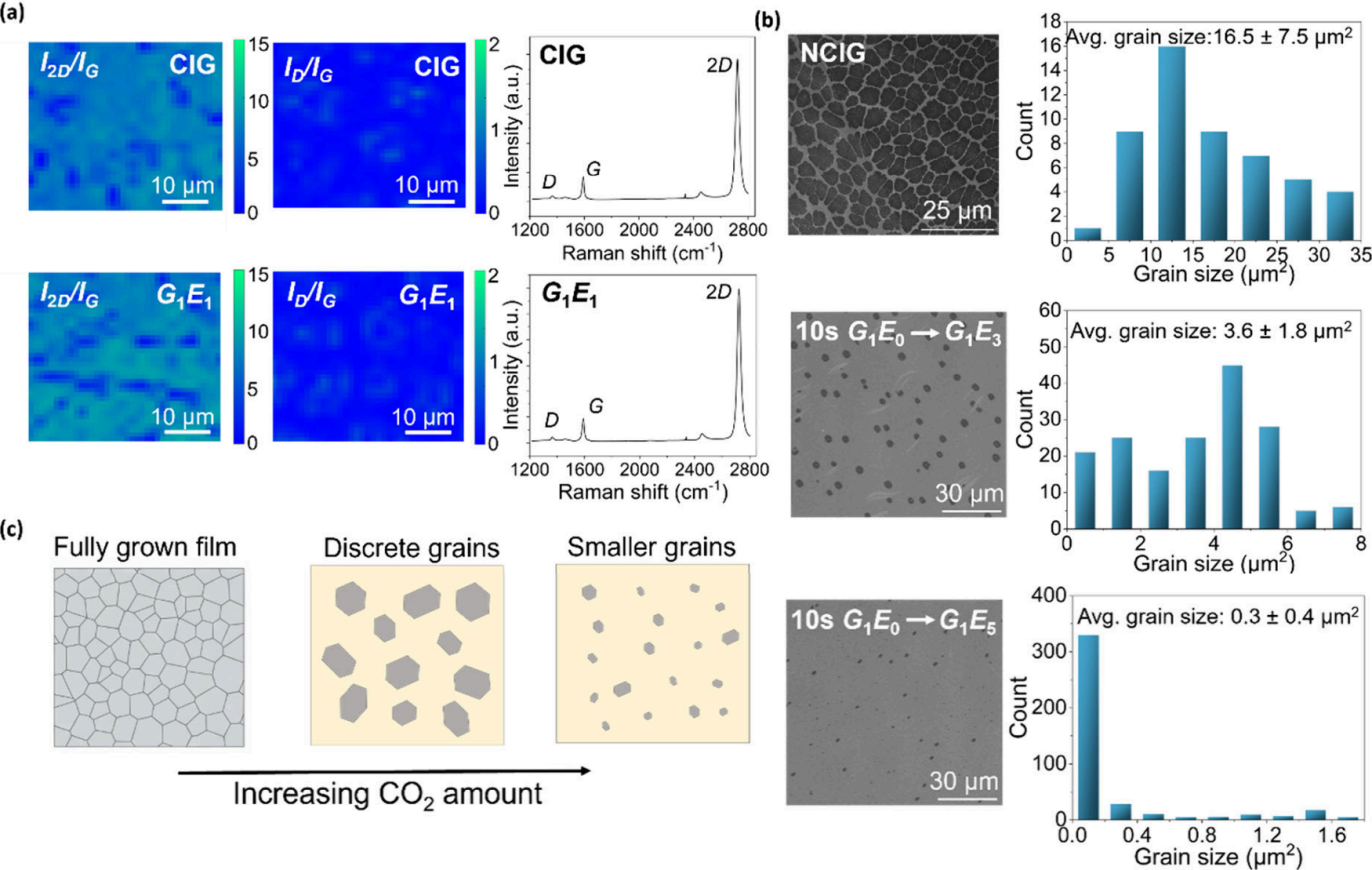
(a) Raman spectroscopy mapping of *I*_2D_/*I*_G_ and *I*_D_/*I*_G_, along with averaged Raman spectra, for graphene synthesized by (i) a 30 min CH_4_ exposure (CIG) and (ii) a 10 s CH_4_ exposure followed by a 30 min exposure to a CH_4_/CO_2_ mixture (G_1_E_1_). (b) SEM images of graphene grown by a 10 s CH_4_ exposure (NCIG), followed by a 30 min exposure to G_1_E_3_ and G_1_E_5_, along with the corresponding grain-size distributions measured by SEM. (c) Effect of CO_2_ on graphene grain intergrowth and morphology.

To understand the bidirectional kinetic regime for pore expansion and contraction in a CO_2_/CH_4_ gas mixture in the CVD environment, NCIG was exposed to a mixed CH_4_/CO_2_ atmosphere for 30 min at 1000 °C. The composition of CH_4_ (growth) and CO_2_ (etchant) is hereafter termed *G*_a_ *E*_b_, where *a* and *b* represent their relative flow rates. In all experiments, the H_2_ flow rate (8 sccm) and total pressure (800 mTorr) were kept constant while the CH_4_ and CO_2_ flow rates were varied accordingly. Thus, *a* and *b* denote the relative amounts of CH_4_ and CO_2_ in the gas mixture; for example, *G*_1_ *E*_0_, *G*_1_ *E*_0.5_, *G*_1_ *E*_1_, *G*_1_ *E*_3_, and *G*_1_ *E*_5_ correspond to CH_4_:CO_2_ ratios of 1:0, 1:0.5, 1:1, 1:3, and 1:5, respectively.

Micro-Raman spectroscopy mapping revealed that under the *G*_1_ *E*_1_ condition, the NCIG film remained continuous with intact intergrown graphene grains, similar to the CIG sample (Figure 1a). Micro-Raman spectroscopy mapping revealed that under the *G*_1_ *E*_1_ condition, the NCIG film remained continuous with intact intergrown graphene grains, similar to the CIG sample (Figure 1a). The isolated NCIG grains were completely intergrown under these conditions (Figure S3 and Figure 1a). Averaged Raman spectra (*n* = 400) collected over a large area further confirmed this, where the defect peak (D peak) was not pronounced. This suggests that the CO_2_ concentration in *G*_1_ *E*_1_ is not high enough, resulting in CVD conditions where the growth rate exceeds the rate of CO_2_-induced etching.

To assess whether the direction of the kinetic regime can be reversed from net growth to net etching, the CO_2_ concentration was progressively increased. When the CO_2_ composition was increased 3-fold to *G*_1_ *E*_3_, a substantial etching of the graphene lattice was observed. The average lateral size of individual grains of NCIG shrank from 16.5 ± 7.5 to 3.6 ± 1.8 μm (Figure 1b). This indicates a transition to a competitive growth–etching regime (from net growth to net etching), in which CO_2_-mediated carbon removal reduces the concentration of active growth precursors. Notably, the grains developed well-defined faceted morphologies (Figure S4). This indicates that lateral grain growth becomes limited by precursor diffusion, and growth proceeds preferentially along energetically favorable crystallographic directions, giving rise to anisotropic, faceted grain shapes.^41^

Further increasing the CO_2_ concentration 5-fold to *G*_1_ *E*_5_ resulted in a pronounced reduction in grain size to 0.3 ± 0.4 μm (Figure 1b). At this stage, etching dominates over growth, leading to the fragmentation of initially well-intergrown graphene films into isolated, nanoscale grains. These experiments indicate that increasing the CO_2_ concentration from *G*_1_ *E*_1_ to *G*_1_ *E*_3_ and *G*_1_ *E*_5_ systematically shifts the balance from growth-dominated to etching-dominated regimes (Figure 1c), establishing that bidirectional growth and etching can be achieved by simply tuning the gas mixture composition. Importantly, CO_2_ does not result in measurable oxidation of the graphene surface, avoiding additional complexities that may arise from oxygen functional groups.^42^ This allows facile experiments for tuning bidirectional growth or etching rates to be set up.

Next, to further fine-tune conditions for controlled pore shrinkage, we narrowed the concentration profile toward controlled grain growth. For this, we first prepared CIG and then incorporated micrometer-sized, faceted pores expanding intrinsic vacancy defects in the film by exposing the film to CO_2_ at 950 °C, following the method reported in the literature (Figure 2a and Figure S5).^43^ Next, the pore shrinkage experiments were performed in the presence of the CH_4_/CO_2_ mixture. The experiments were intentionally performed at a lower temperature (800 °C) to slow the kinetics and access incompletely grown domains for characterization.

**Figure 2:**
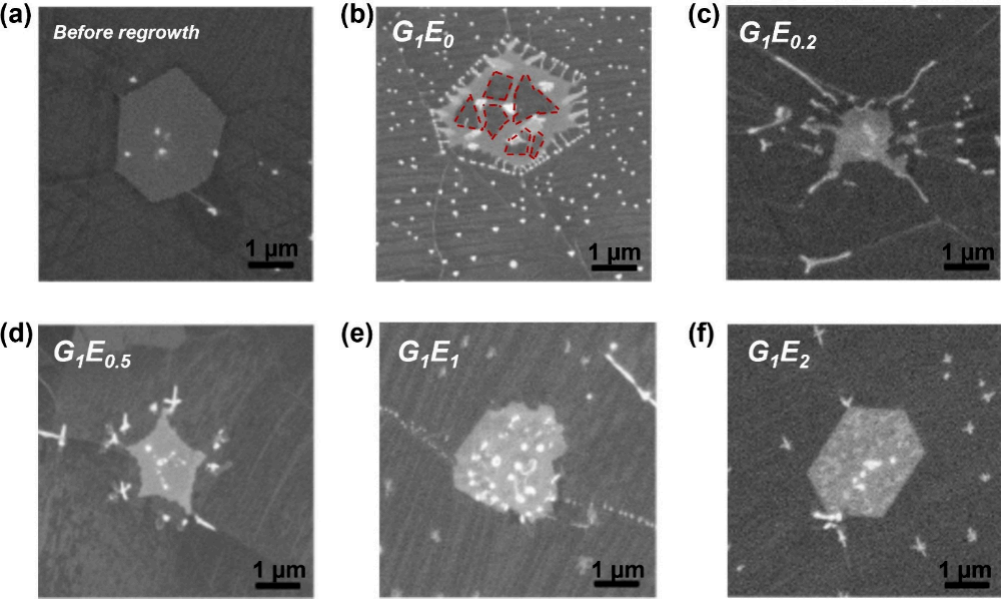
SEM images of the micrometer-scale pore in CIG. (a) As-synthesized pore upon exposure of CIG to CO_2_. SEM images after pore shrinkage by exposure under (b) *G*_1_ *E*_0_, (c) *G*_1_ *E*_0.2_, (d) *G*_1_ *E*_0.5_, (e) *G*_1_ *E*_1_, and (f) *G*_1_ *E*_2_ conditions for 15 min. The red dashed lines in panel b indicate graphene domains nucleated inside the pore.

In the absence of CO_2_ (*G*_1_ *E*_0_), growth was observed both at the pore edge and inside the pores. The latter are new graphene domains nucleated on the exposed Cu surface within the pores (Figure 2b). The number of nucleation sites increased with the size of the exposed Cu area (Figure S6). This is as expected, given that a larger exposed area increases the probability of nucleation. Interestingly, the introduction of CO_2_ at concentrations as low as *G*_1_ *E*_0.2_ was sufficient to completely suppress graphene nucleation (Figure 2c–f). Graphene nucleation typically occurs when active carbon species reach a critical concentration on the Cu substrate.^44^ As shown above, CO_2_ prevents the localized supersaturation of active carbon species, thereby inhibiting graphene nucleation on the metal surface. This indicates the active role of CO_2_ in reducing the population of active growth species, which would otherwise nucleate graphene domains.

While we observed graphene growth along the edge for CO_2_ concentrations up to *G*_1_ *E*_1_, it was suppressed for higher CO_2_ concentrations (*G*_1_ *E*_2_ (Figure 2f)), where pores maintained their faceted shape. This indicates that the shrinkage of graphene pores could be regulated progressively from *G*_1_ *E*_0_ (no CO_2_) to *G*_1_ *E*_1_, where a further increase in the CO_2_ concentration to *G*_1_ *E*_2_ ruled out pore shrinkage (Figure 2f). This behavior reflects the competition between CH_4_-mediated carbon addition and CO_2_-induced carbon removal. At low CO_2_ concentrations, growth dominates, leading to active edge reconstruction and irregular kinetically driven morphologies. As the CO_2_ concentration increases, etching begins to counterbalance growth, resulting in a more controlled edge evolution. At high CO_2_ concentrations, the etching rate is sufficiently strong to suppress CH_4_-driven incorporation, leading to a minimal net edge advancement. Consequently, the pores preserve their initial faceted geometry rather than evolve through continued growth or etching (Figure S6).

To investigate the roles of CH_4_ and CO_2_ in the growth of graphene domains, carbon isotope labeling combined with Raman spectroscopy was used. Raman spectra of graphene synthesized with ^13^CH_4_ revealed an ∼100 cm^–1^ red-shift in the 2*D* peak position, compared to that synthesized with ^12^CH_4_, corresponding to the inverse relationship between the Raman mode frequency and atomic mass (Note S1), which is consistent with the literature. This was then used to identify the source of graphene growth in subsequent experiments.

In a two-step experiment, graphene was first synthesized using ^12^CH_4_, after which micrometer-sized pores were etched using CO_2_, as described in the previous section (Figure 3a­(i)). Regrowth of graphene domains in the vicinity of these pores was then performed with ^13^CH_4_ under *G*_1_ *E*_0_ and *G*_1_ *E*_0.5_ conditions. Representative SEM images of pores after the regrowth process are shown in panels ii and iii of Figure 3a.

**Figure 3:**
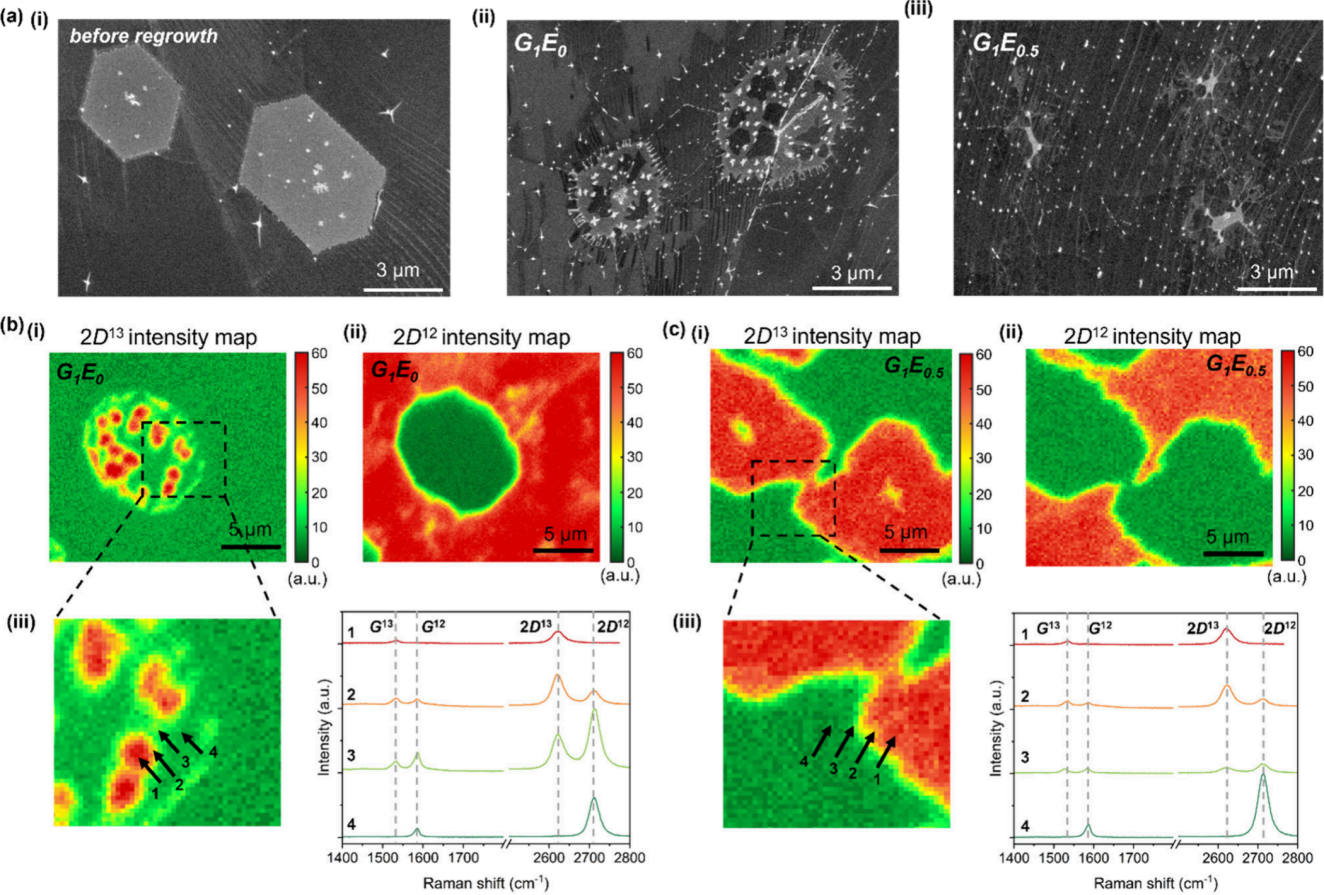
(a) SEM images after pore shrinkage by exposure under (i) before regrowth, (ii) *G*_1_ *E*_0_, and (iii) *G*_1_ *E*_0.5_ conditions at 800 °C. (b and c) Isotope-resolved Raman maps of (i) 2*D* ^13^ and (ii) 2*D* ^12^ peak intensity for the *G*_1_ *E*_0_ and *G*_1_ *E*_0.5_ conditions, showing signals from both ^13^C and ^12^C in panels i and ii, respectively. In panel iii, enlarged Raman maps of the 2*D* ^13^ peak intensity under (b) *G*_1_ *E*_0_ and (c) *G*_1_ *E*_0.5_ conditions, along with representative Raman spectra at positions marked 1–4 in panels iii.

Isotope-resolved Raman mapping and corresponding spectra revealed the emergence of a pronounced 2*D* ^13^ peak inside pores (marked with arrow 1), indicating that newly formed graphene domains during regrowth originated exclusively from ^13^CH_4_ rather than from residual carbon species in the reactor or from carbon-containing intermediates derived from CO_2_ (Figure 3b,c). This confirms that under these conditions, carbon in CO_2_ did not participate in graphene growth and that CO_2_ was not reduced to carbon precursors in the presence of H_2_. Consequently, the role of CO_2_ is limited to that of a selective etchant, rather than a carbon source.

At pore edges (marked by arrows 2 and 3), mixed ^12^C/^13^C Raman signatures were observed due to the laser spot size (∼200 nm) overlapping regions of both original and regrown graphene. The intensity of the 2*D* ^12^ and 2*D* ^13^ peaks corresponds to the proportion of the laser spot area occupied by each carbon isotope.^45^ In this way, the spectra of freshly nucleated graphene matched the spectra of that synthesized with ^13^CH_4_, while areas near the pore edges contained a mixture of ^12^C and ^13^C isotopes.

Importantly, Raman mapping revealed a clear distinction in pore shrinkage behavior across different gas environments. In the absence of CO_2_ (*G*_1_ *E*_0_), new nucleation sites appeared within the pores, and their areal coverage increased with a prolonged growth time. In contrast, the introduction of CO_2_ (*G*_1_ *E*_0.5_) completely suppressed nucleation within the pore interior, with regrowth confined exclusively to pore edges, which is consistent with SEM observations in panels ii and iii of Figure 3a. This suppression of in-pore nucleation highlights the critical role of CO_2_ in removing active growth precursors and preventing nucleation inside pores, thereby selectively initiating growth from the graphene pore edges.

Motivated by findings on the lattice reconstruction under a CH_4_/CO_2_ atmosphere, we then pursued the shrinkage of nanometer-scale graphene pores to angstrom-scale pores, which is needed to achieve gas-selective transport. A schematic representation of the proposed pore-size control with lattice reconstruction in the presence of CO_2_ is shown in Figure 4a.

**Figure 4:**
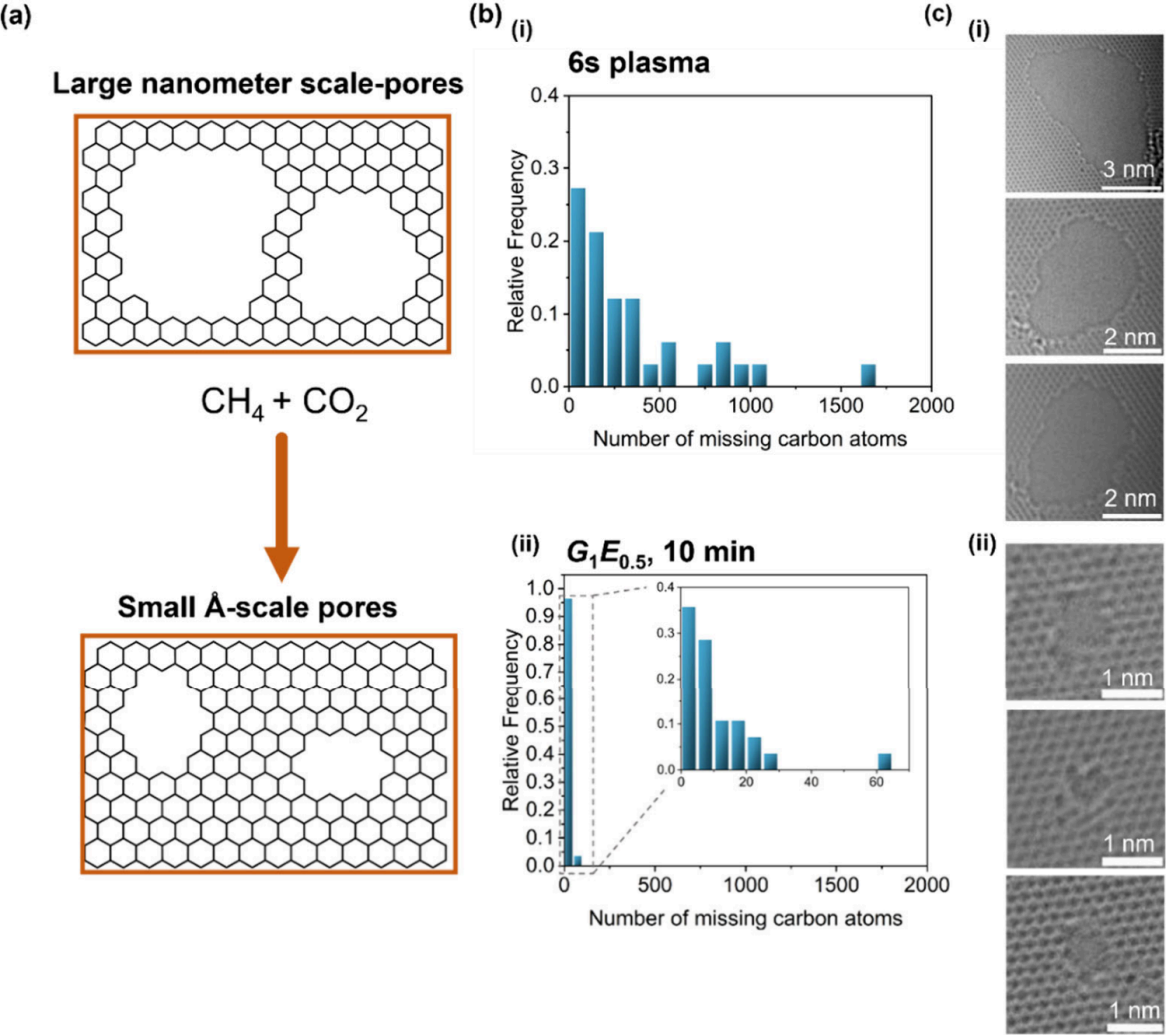
(a) Schematic of the PSD in the pore shrinkage process in the presence of both CH_4_ and CO_2_. (b) Corresponding PSD for (i) porous graphene after a 6 s plasma exposure and (ii) a sample exposed to the *G*_1_ *E*_0.5_ condition for 10 min. (c) AC-HRTEM images of porous graphene (i) prepared by a 6 s plasma treatment and (ii) after a 10 min exposure to the *G*_1_ *E*_0.5_ condition for pore shrinkage.

To directly visualize changes in PSD, we used aberration-corrected high-resolution transmission electron microscopy (AC-HRTEM). As-synthesized graphene samples were subjected to a 6 s O_2_ plasma treatment to intentionally introduce large nanometer-scale pores. The samples exhibited a broad PSD with a mean pore area of 11.3 ± 6 nm^2^ and occasional larger pores with an area of up to ∼28 nm^2^, as illustrated in representative AC-HRTEM images in Figure 4b.

The plasma-treated graphene samples were subsequently subjected to a pore shrinkage process under the *G*_1_ *E*_0.5_ conditions at 800 °C for 10 min. Following this, a dramatic reduction in pore size was observed. In nanometer-scale pores, the growth is significantly slower than in micrometer-scale pores due to the limited availability of the catalytic Cu surface required for CH_4_ decomposition and carbon supply.^46^ In addition, the CO_2_ etching rate also decreases at smaller pore sizes.^43^ By comparing AC-HRTEM (for nanometer-scale pores) and SEM (for micrometer-scale pores) images, we roughly estimate that pore evolution in nanometer-scale pores is approximately 2 orders of magnitude slower (∼0.3 nm min^–1^) than in micrometer-scale pores (∼30 nm min^–1^).

Regardless, pore-size reduction was significant. Specifically, the average number of missing carbon atoms per pore decreased by nearly 95%, from ∼340 to 10, corresponding to a size suitable for gas-selective transport.^47^ The average pore size significantly decreased from 11.3 ± 6 to 0.41 ± 0.3 nm^2^ after the shrinkage treatment. This shrinkage process resulted in a pronounced narrowing of the PSD, accompanied by a substantial suppression of the long-tail distribution, as shown in Figure 4b. Importantly, the pore density remained essentially unchanged ((0.9 ± 0.8) × 10^11^ and (1 ± 0.9) × 10^11^ pores/cm^2^ before and after shrinkage, respectively), indicating that the process selectively reduces pore size without affecting pore population. These observations clearly demonstrate that pore shrinkage in the presence of CH_4_ and CO_2_ effectively contracts existing pores and sharpens the PSD, two key attributes for enhancing molecular sieving and gas separation selectivity.

Crucially, AC-HRTEM analysis revealed that the newly formed graphene at the pore edges preserved the crystallographic orientation of the surrounding lattice. No grain boundaries or rotational mismatches were observed near the regrown pore edges, confirming edge-mediated lattice reconstruction rather than random carbon deposition. This structural continuity suggests that CO_2_ acts as a selective etchant, preferentially removing undercoordinated or defective carbon atoms at the pore edge, while CH_4_ serves as a carbon precursor that promotes the reconstruction of energetically favorable sp^2^-bonded graphene. Therefore, an optimized regrowth process to achieve angstrom-scale pores with a narrow PSD could be achieved, with clear evidence of growth via edge-mediated lattice reconstruction.

We next evaluated how pore-size tuning via CH_4_/CO_2_ exposure translates into gas separation performance. To fabricate membranes, single-layer graphene films resting on Cu foil were exposed to O_2_ plasma for 6 s to create a porous structure, as discussed in the previous section, yielding large (11.3 ± 6 nm^2^) nonselective pores. The resulting samples were grown under varying CH_4_/CO_2_ ratios. Following this, the samples were coated with a mechanical reinforcing film (MRF) of polytrimethylsilylpropyne (PTMSP) and subsequently transferred onto a porous support for gas permeation testing (see the detailed preparation protocol in the Methods).

Single-gas permeation tests were conducted using H_2_ and C_3_H_8_. As expected, as-synthesized porous graphene after a 6 s plasma exposure exhibited negligible gas selectivity, consistent with the nonselective nature of nanometer-sized pores. Upon pore shrinkage under various CH_4_/CO_2_ growth environments, the H_2_/C_3_H_8_ selectivity increased noticeably (Figure 5a). The highest gas selectivity was observed at 10 min of *G*_1_ *E*_0.5_, with the H_2_/C_3_H_8_ ideal selectivity reaching 26. This significant increase in H_2_/C_3_H_8_ selectivity indicates the effective reduction of nonselective pores and the refinement of the PSD into the molecular sieving regime. Further increases in the CO_2_ concentration during pore-size tuning (*G*_1_ *E*_1_) led to a decrease in selectivity, indicating insufficient pore shrinkage.

**Figure 5:**
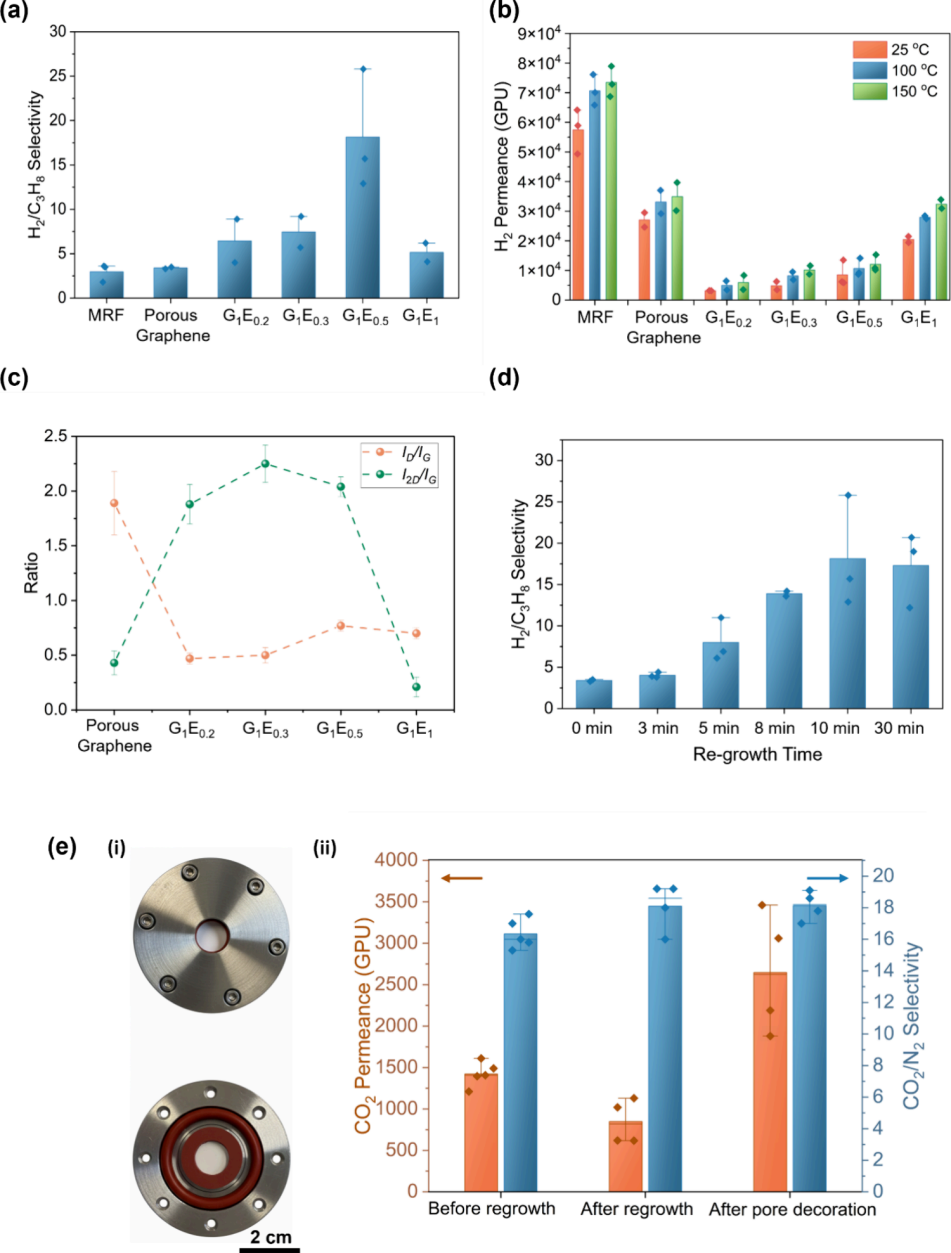
(a) H_2_/C_3_H_8_ ideal gas selectivity as a function of CH_4_/CO_2_ ratio, for a 10 min exposure during PSD tuning. (b) Trend of H_2_ permeance with an increase in the amount of CO_2_ during pore shrinkage for a 10 min exposure, measured at 25, 100, and 150 °C. (c) Raman spectroscopy metrics (*I*_2D_/*I*_G_ and *I*_D_/*I*_G_ ratios) highlighting changes in graphene quality across CH_4_/CO_2_ conditions for a 10 min exposure. (d) Effect of regrowth duration on H_2_/C_3_H_8_ ideal gas selectivity. (e) (i) Photographs of centimeter-scale membrane modules and (ii) CO_2_/N_2_ separation performance at room temperature of centimeter-scale graphene membranes before^31^ and after pore shrinkage, and after pore decoration.

As shown in Figure 5b, H_2_ permeance exhibits a nonmonotonic trend with an increase in CO_2_ concentration during PSD tuning under 10 min exposure. It initially decreases due to pore-size contraction but increases again beyond the *G*_1_ *E*_0.2_ condition. This behavior reflects the evolving balance between CH_4_-driven growth and CO_2_-induced etching. At low CO_2_ concentrations (e.g., *G*_1_ *E*_0.2_ and *G*_1_ *E*_0.3_), CH_4_-driven growth dominates, leading to rapid edge-mediated lattice reconstruction. However, this can also promote uncontrolled carbon deposition or partial pore closure, resulting in a PSD that is not optimally refined for molecular sieving, despite being in the contraction regime.

With an increasing CO_2_ concentration (e.g., *G*_1_ *E*_0.5_), etching begins to counterbalance CH_4_-driven growth. This suppresses excessive carbon deposition and prevents uncontrolled pore closure or nucleation while still allowing controlled edge-mediated reconstruction to proceed. As a result, pore shrinkage becomes more regulated. This shift explains the increase in H_2_ permeance beyond *G*_1_ *E*_0.2_, as the pore sizes are more effectively tuned for selective gas transport.

This behavior is consistent with Raman spectroscopy results (Figure 5c). From *G*_1_ *E*_0_ to *G*_1_ *E*_0.3_, the *I*_2D_/*I*_G_ ratio increases and the *I*_D_/*I*_G_ ratio decreases, indicating improved graphene crystallinity and reduced disorder under growth-dominated conditions. The *I*_D_/*I*_G_ ratio is widely used as a measure of defect density in graphene, with lower values corresponding to fewer defects and a more intact sp^2^ lattice. Concurrently, the increase in *I*_2D_/*I*_G_ reflects enhanced lattice ordering and improved electronic coherence of the graphene domains.^48,49^ Together, these trends suggest that CH_4_-driven carbon incorporation promotes healing of defect sites and partial reconstruction of the graphene lattice, leading to reduced vacancy concentration and improved structural quality.

However, this trend is reversed at *G*_1_ *E*_0.5_, consistent with a transition toward a balanced growth–etching regime. In parallel, the average H_2_ permeance increases significantly from 4861 to 9687 GPU when moving from *G*_1_ *E*_0.3_ to *G*_1_ *E*_0.5_, while maintaining low C_3_H_8_ permeance (740 and 638 GPU, respectively), reflecting enhanced molecular sieving performance. At higher CO_2_ concentrations (e.g., *G*_1_ *E*_1_), etching becomes dominant, leading to pore expansion and a loss of selectivity. Therefore, *G*_1_ *E*_0.5_ represents an optimal condition where CO_2_ effectively regulates the growth–etching balance to produce highly selective angstrom-scale pores. The ability to both shrink and expand pores through this postsynthetic treatment, providing bidirectional pore-size tuning, is highly attractive, as it will allow one to address a diverse set of separation challenges in the future.

The regrowth duration also played a crucial role in tuning separation performance. H_2_/C_3_H_8_ selectivity improved steadily with an increase in regrowth time, reaching an average of 18 at 10 min (Figure 5d). Beyond this duration, selectivity did not increase, indicating that a dynamic balance between pore shrinkage and expansion was reached under these conditions. Further increasing the regrowth time led to a noticeable decrease in H_2_ permeance (Figure S8), indicating that prolonged exposure does not simply refine the pore-size distribution but instead introduces additional resistance to gas transport. This behavior can be attributed to excessive pore shrinkage or deposition of an additional carbon layer. Such deposition can arise from gas-phase carbon species and the reduced catalytic activity of the Cu substrate under prolonged exposure, leading to the formation and adsorption of carbon clusters.^50,51^ The accumulation of these species can partially block or constrict pore openings, further limiting permeance without improving selectivity. Furthermore, at longer time scales, as pores approach the subnanometer regime, further shrinkage becomes limited due to steric constraints on precursor access and reduced edge reactivity. A comparison of single-layer graphene membrane performance for H_2_/C_3_H_8_ separation performance after pore shrinkage with state-of-the-art membranes is provided in Table S1.

After identifying optimal conditions for enhanced gas selectivity (*G*_1_ *E*_0.5_ for 10 min), we extended this pore shrinkage strategy to centimeter-scale membranes to improve CO_2_/N_2_ separation performance. Large-area (∼10 cm^2^) porous, CO_2_-selective graphene membranes were first prepared using room-temperature O_3_ treatment followed by photonic gasification.^31^ This approach yielded membranes with an average CO_2_ permeance of ∼1400 GPU and a CO_2_/N_2_ selectivity of 16. For subsequent pore shrinkage, O_3_-treated samples were annealed at 800 °C under H_2_ and then exposed to the G_1_E_0.5_ condition for 10 min. The treated graphene coupons were subsequently coated with MRF, transferred onto porous supports, and assembled into membrane modules with a 1 cm diameter. Photographs of representative membrane modules are shown in panel i of Figure 5e. Following pore shrinkage, the membranes exhibited a modest decrease in CO_2_ permeance and an increase in CO_2_/N_2_ selectivity as per expectations from the pore shrinkage experiment (Figure 5e­(ii)). Such a treatment under a H_2_ atmosphere is expected to reduce the population of the O functional group at the pore edge.

Since O-functionalized pores are advantageous for CO_2_ permeation,^52^ a brief (3 min) ozone treatment was performed at room temperature. This step was motivated by prior studies demonstrating that short ozone exposure can functionalize graphene pore edges.^31,53^ In our system, this treatment introduces oxygen-containing functional groups that enhance CO_2‑_pore interactions and facilitate CO_2_ transport. At the same time, slight pore edge modification or limited expansion may also occur during ozone exposure of porous graphene on Cu due to an increased reactivity of pore edges on Cu.^53^ As a result, CO_2_ permeance increases significantly from 1136 ± 267 to 2647 ± 736 GPU, while CO_2_/N_2_ selectivity is largely preserved (≈18 (Figure 5e­(ii)). This highlights the combined effect of pore functionalization and subtle structural modification and underscores the utility of the pore-size-tuning method for carbon capture applications. A comparison of single-layer graphene membranes with state-of-the-art membranes is presented in Table S2.

Overall, we demonstrated a bidirectional strategy for pore-size modulation in graphene through the controlled interplay of CH_4_ and CO_2_ as growth and etching agents, respectively. By tuning the CH_4_/CO_2_ ratio, we achieved kinetic control over the crystallization and etching dynamics at the graphene pore edge, enabling both pore expansion and contraction. Isotope labeling combined with Raman spectroscopy confirmed that carbon for regrowth originates solely from CH_4_, with no contribution from CO_2_ or residual carbon. High-resolution microscopy showed edge-mediated lattice reconstruction, preserving lattice orientation. These results highlight the role of CH_4_/CO_2_ dynamics in controlling porosity and demonstrate the method’s potential for scalable fabrication of high-quality, selectively porous graphene. This work establishes a robust and versatile framework for engineering PSD in graphene with angstrom precision. This approach opens a viable pathway for advancing the development of high-performance graphene-based membranes for gas separation and other nanoscale transport applications.

## Supplementary Material



## Notes

Additional experimental procedures, microscopy images, membrane separation performance plot, experimental setup drawings and photographs, and tables comparing membrane performance (PDF)
