# Supplementary material for: Bidirectional Size Control for Angstrom-Scale Graphene Pores by Competitive Growth and Etching
Source: Nano Lett. 2026 May 8;26(23):7612–20. doi: 10.1021/acs.nanolett.6c01120 (PMC13281700; doi:10.1021/acs.nanolett.6c01120)
Supplement: Supplementary file 1 [file nl6c01120_si_001.pdf]

Supporting Information

Bidirectional Size Control for Ångström-Scale Graphene Pores by Competitive Growth and Etching

Ceren Kocaman‡ [a], Mojtaba Chevalier‡ [a], Yueqing Shen[a], Luis Francisco Villalobos[a, b], and Kumar Varoon Agrawal[a].*

[a]Prof., K.V, Agrawal; Dr., C, Kocaman; M, Chevalier; Y, Shen

Laboratory of Advanced Separations (LAS)
École Polytechnique Fédérale de Lausanne (EPFL)
Rue de l'Industrie 17, 1950 Sion, Switzerland
E-mail: kumar.agrawal@epfl.ch

[b] Prof. L.F, Villalobos
Chemical Engineering and Materials Science
USC Viterbi School of Engineering
3650 McClintock Avenue, OHE 106
Los Angeles, CA

Table of Contents

1.	Experimental procedures	S1
2.	Characterization procedures	S2
3.	Additional figures	S3
4.	Additional tables	S4
5.	Additional notes	S5
6.	References	S6

1. Experimental Procedures

1.1. Graphene Synthesis

Single-layer graphene was synthesized on Alfa Aesar Cu foil (99.8% purity) using a low-pressure chemical vapor deposition (LPCVD) method. The schematic of CVD system is shown in Figure S7. Initially, the foil was sonicated in isopropanol for 5 minutes, then placed inside a CVD reactor equipped with a quartz tube. The Cu foil underwent high-temperature annealing before graphene synthesis to reduce surface roughness and remove potential contaminants.[1] The cleaning process involved annealing the Cu foil in a 760 Torr CO_2 environment at 1000 °C for 30 minutes. Following this, the reactor was filled with a 760 Torr mixture of H_2 and Ar (1:10 ratio), and the sample was heated to 1075 °C, where it was maintained for 1 hour. The temperature was then gradually lowered to 1000 °C followed by an additional 30 minutes of H_2 annealing at 80 mTorr (H_2 flow of 8 sccm). Graphene synthesis was initiated by introducing 24 sccm of CH_4 into the system for 30 minutes (total system pressure of 460 mTorr). Finally, while maintaining the H_2 flow, the sample was rapidly cooled to room temperature.

1.2. Pore Formation and Expansion

To incorporate pores on the graphene lattice, O_2-plasma treatment was carried out on graphene samples by a13.56 MHz MTI EQ-PCE-3 plasma generator at 17 W. The chamber was fully evacuated and purged with O_2 three times before stabilizing the system at 50 mTorr by controlling the O_2 flow. Then, plasma was generated for the desired duration to introduce pores in the graphene lattice.

To expand intrinsic defects and generate micrometer-sized pores, CO_2 etching was carried out inside the same CVD reactor used for graphene synthesis. The quartz tube connections to the vacuum and gas inlet systems were enclosed within cylinders with a pure N_2 flow to prevent O_2 leakage from the environment into the CVD reactor. CO_2 gas was passed through ultraclean gas filters from PerkinElmer to eliminate O_2 impurities.

The porous graphene on Cu foil sample was reloaded into the CVD reactor. The sample was heated to 1000 °C in an H_2 environment and maintained at this temperature for 3 minutes to stabilize the system before introducing CO_2 for pore expansion. 500 mTorr CO_2 alongside 300 mTorr H_2 were introduced into the reactor for pore expansion. Finally, after the desired etching duration, the sample was removed from the heating zone to stop the reaction.

To prepare centimeter-scale CO_2-selective membranes, a room temperature O_3 treatment followed by photonic gasification was employed.[2] The samples were placed in a custom-made flow channel reactor inside a tubular ozone chamber, and exposed to 100 sccm of a 9.35 mol% O_3/O_2 gas mixture for 1 hour. Following the ozone treatment, the oxidized samples underwent lattice gasification by exposure to 390 nm (3.2 eV) light for 5 s.

1.3. Pore Regrowth by CO_2-CH_4

Pore edge regrowth was carried out inside the same CVD reactor used during the previous steps. For samples etched with CO_2, after the etching process, the samples were cooled and maintained at 800 °C for 3 minutes in an H_2 environment to ensure a uniform temperature profile. Following this, CH_4 and CO_2 at varying ratios, along with 185 mTorr H_2, were introduced into the reactor for the desired duration.

For O_2 plasma and O_3 -treated samples, the system was first evacuated to < 1 mTorr and purged with H_2 four times after placing the samples inside the CVD reactor. The samples were then heated to 600 °C and held at this temperature for 30 minutes in 760 Torr H_2 to remove surface contaminants introduced during the plasma treatment process. Subsequently, the system was heated to 800 °C and maintained at this temperature for 3 minutes before performing regrowth experiments. To suppress silica (SiO_2)

contamination released from the CVD quartz tube during high-temperature processing, graphene samples were placed inside a Cu sheath (Figure S9) for membrane preparation.

1.4. Pore decoration by mild O_3 treatment

After pore regrowth by CO_2-CH_4 exposure, the samples were treated with ozone at room temperature for 3 and 15 minutes. The samples were placed in a tubular ozone chamber and exposed to a 9.35 mol% O_3/O_2 gas mixture for the specified duration.

1.5. Membrane Preparation

For membrane preparation, PTMSP layer was used as the mechanical support to prevent cracks and tears in the graphene layer during the graphene transfer process from Cu foil to the tungsten (W) support. For this, 1.25% wt PTMSP in toluene solution was spin-coated on graphene at 1000 rpm for 1 min, and dried overnight at ambient conditions and then in vacuum at 30 ^{o}C for 12 hours. Following that, the underlying Cu support was etched away using aqueous 1 M $FeCl_3$ solution, and the graphene/PTMSP sample was cleaned with 10% wt. HCl solution before rinsing with deionized water three times. Finally, the floating Graphene/PTMSP film was scooped with a porous tungsten (W) support. Optical images of the support and PTMSP-supported porous graphene membrane on the W support is shown in Figure S11.

For centimeter-scale membranes, graphene samples coated with 3 wt% PTMSP were detached from their Cu support via electrochemical delamination and rinsed with water. The samples were then transferred onto a porous polymeric support (PES with 0.2–0.8 µm pore size) and dried overnight at room temperature.

1.6. Gas Permeation Tests

Gas permeation tests were performed in a homemade permeation setup consisting of a leak-tight membrane module. Briefly, the porous W supporting the graphene membrane acted as a gasket between Swagelok VCR fittings, and the setup was kept inside a temperature-controlled oven. Ar at 1 bar was used as the sweep gas, and the feed gas was kept at 2 bar. The single-gas permeation measurements

were carried out for H_2, and C_3H_8 gases. A pre-calibrated Hiden Analytical HPR-20 mass spectrometer was used to analyze the permeate stream. Membranes were heated to and maintained at 150 °C to desorb the atmospheric surface contaminants before measuring their permeance. A schematic of the membranes testing setup is shown in Figure S12.

For centimeter-scale membranes, a custom-made single component permeation setups with pure CO_2 and N_2 was used. Membranes were tested at room temperature at 2 bar feed pressure.

2. Characterization Procedures

SEM images were acquired by FEI Teneo scanning electron microscope at an operating voltage of 1 – 2 kV, an operating current of 25 pA, and a working distance of 5 – 7 mm. In-column T3 secondary-electron detector was used to take the high-contrast graphene-Cu surface images. ImageJ was used to analyze the SEM images.

Raman spectroscopy was done by Renishaw inVia™ confocal spectroscope equipped with a 457 nm excitation laser and ZEISS Plan-Apochromat 63X/1.4 Oil DIC objective. Samples were first transferred onto SiO2/Si substrates using the common PMMA transfer method.[3] More than 10000 spectra were collected for each map. The 2D peak intensity and I_D/I_G and I_{2D}/I_G ratios were calculated by subtracting the background and curve fitting the 2*D*, *G*, and *D* peaks in MATLAB.

For AC-HRTEM, samples were prepared by the lacey-carbon assisted film transfer method.[4] The characterization process was conducted with a double-corrected Titan Themis 60-300 (FEI), which features a Wien-type monochromator. To minimize electron radiation damage, an 80 keV incident electron beam was used. The beam was monochromated to mitigate chromatic aberration, and a negative spherical aberration (Cs) of approximately 18–21 μm, along with slight over-focusing, was applied to enhance "bright atom" contrast in the images.

During focusing and imaging, the lattice was subjected to a total electron dose of approximately 2×10^5 e^- Å^{-2}. If necessary, a band-pass filter was applied to improve the visibility of the lattice structure. ImageJ software was used for analyzing AC-HRTEM images, including the measurement of pore size.

The pore sizes were quantified using a graphical method previously developed by our group, in which the number of missing carbon atoms is determined from the measured vacancy area in AC-HRTEM images, using the atomic density of pristine graphene as a reference.[5] Accordingly, all pore sizes were derived from the number of missing carbon atoms rather than direct geometric measurement, ensuring consistency across both nanometer- and Å-scale pores.

For pore size distribution and pore density analysis a total of 42 images were acquired from multiple regions across independently prepared graphene samples to ensure representative sampling. Each image, with an area of approximately 1260 nm^2, was treated as an independent sampling unit.

Pores were identified and counted based on clearly resolved vacancy structures, with only pores containing more than 10 missing carbon atoms included in the pore density analysis. To minimize artifacts, pores overlapping with surface contamination or imaging noise were excluded. The pore density for each image was calculated as the number of pores per unit area and subsequently converted to units of cm^{-2}.

3. Additional Figures

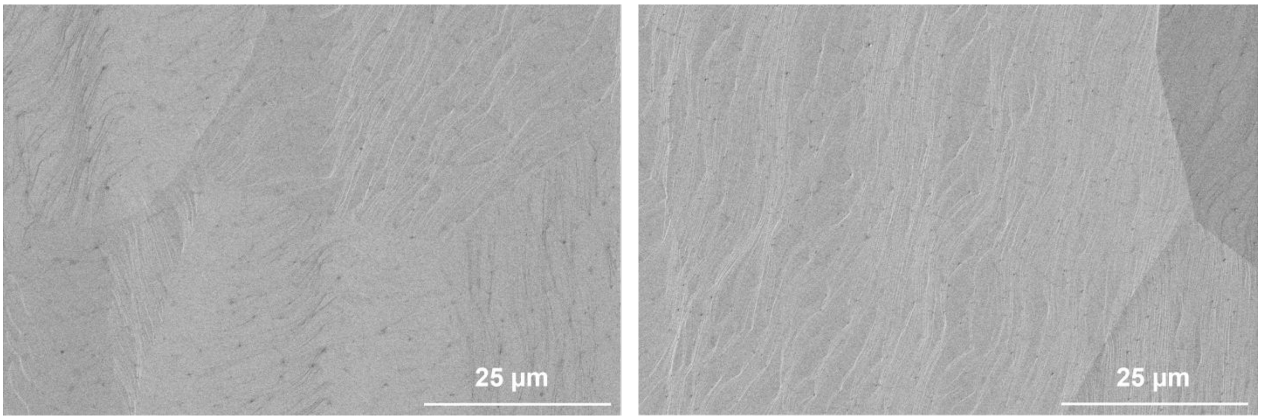

Figure S1. SEM images of completely-intergrown graphene (CIG), synthesized by exposing Cu to CH_4 for 30 min at 1000 °C in the absence of CO_2.

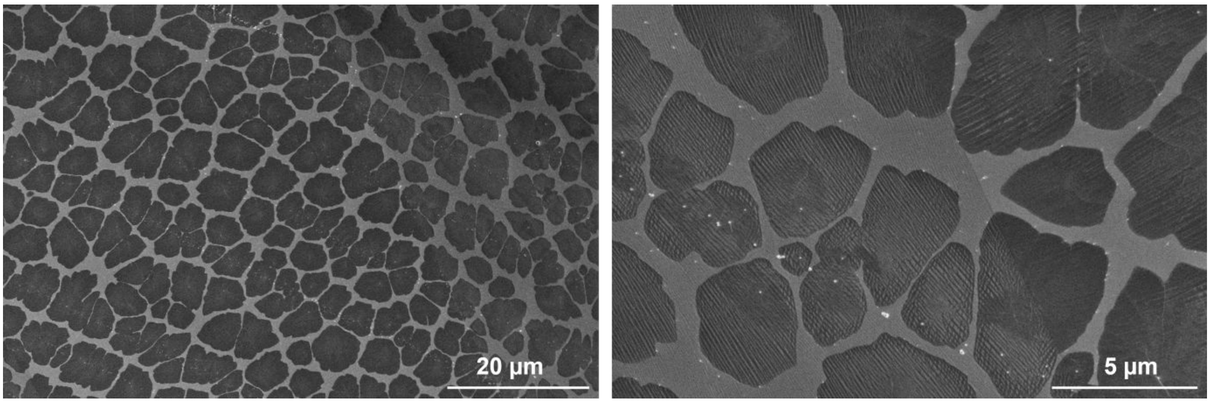

Figure S2. SEM images of not completely intergrown graphene (NCIG), synthesized by exposing Cu foil to the G_1E_0 condition for 10 s at 1000 ºC

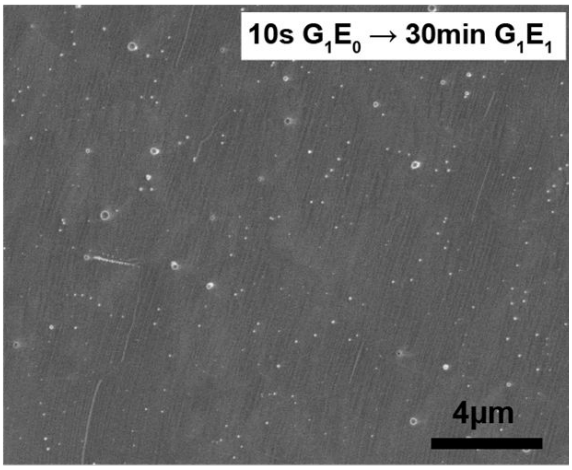

Figure S3. SEM images of not completely intergrown graphene (NCIG, synthesized by G_1E_0 condition for 10 s), followed by exposure to G_1E_1 condition for 30 min at 1000 ºC

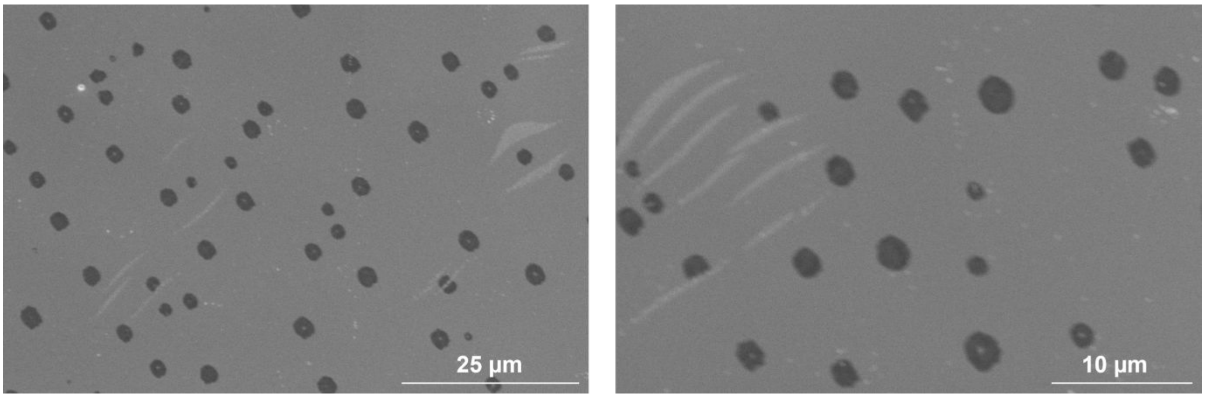

Figure S4. SEM images of graphene grown by 10 s CH_4 exposure followed by 30 min exposure to G_1E_3.

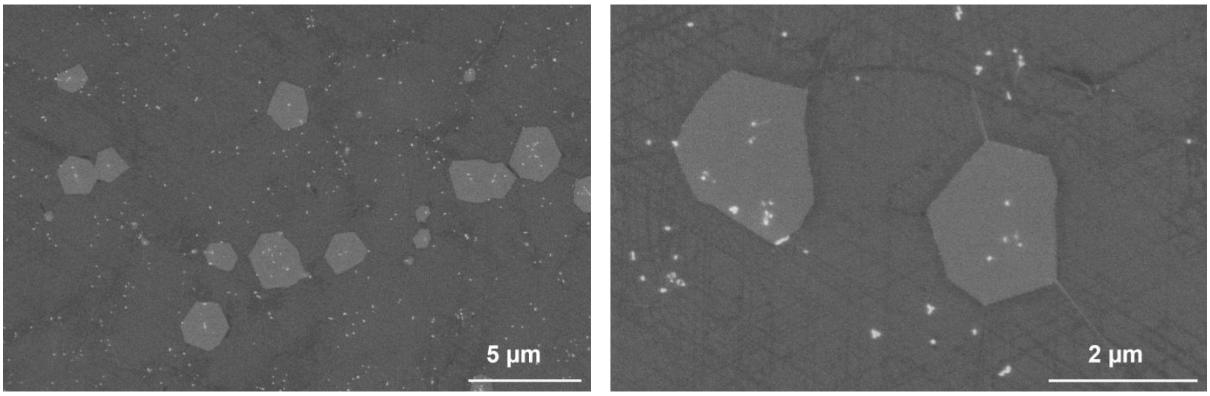

Figure S5. SEM images of incorporated micrometer-sized, faceted pores formed by expanding intrinsic vacancy defects in the graphene film by exposing the film to CO_2 at 950 °C.

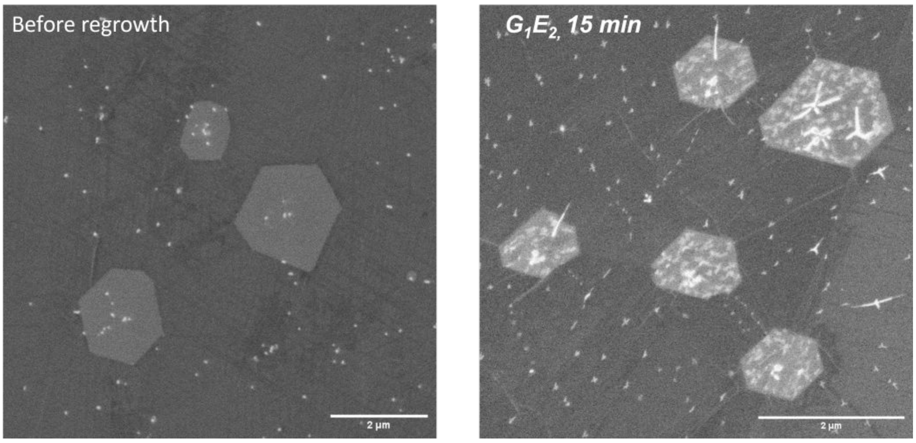

Figure S6. SEM images of incorporated micrometer-sized, before and after regrowth experiments with G_1E_2 condition for 15 min.

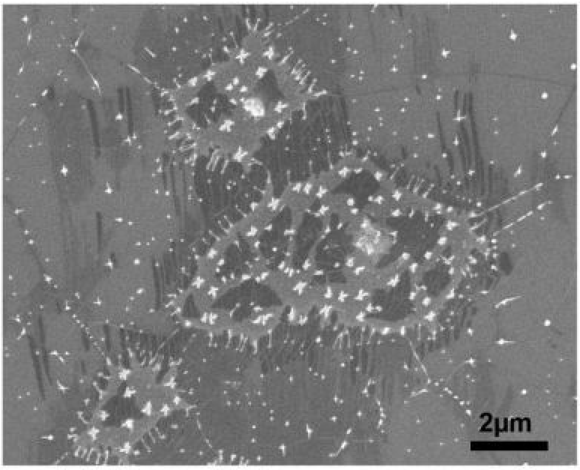

Figure S7. SEM image of the SLG on the Cu foil after exposing to the G_1E_0 regrowth condition at 800 °C for 15 min, showcasing exposed-Cu-area dependent graphene nucleation

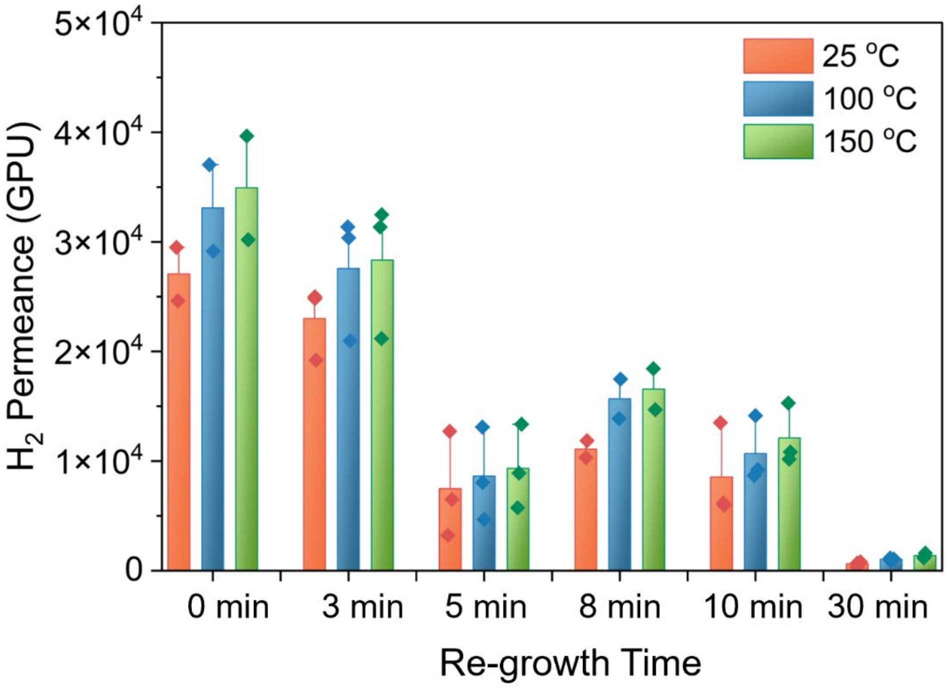

Figure S8. H_2 permeance trend with increasing regrowth time, during pore shrinkage under $G_1E_{0.5}$, measured at 25, 100 and 150°C.

(a)

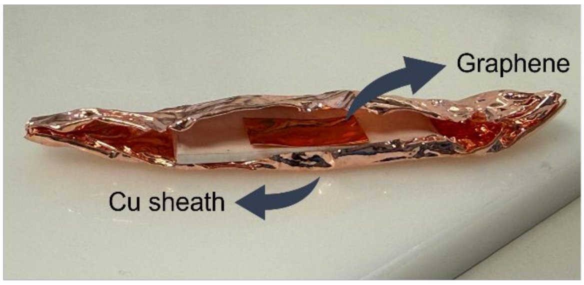

(b)

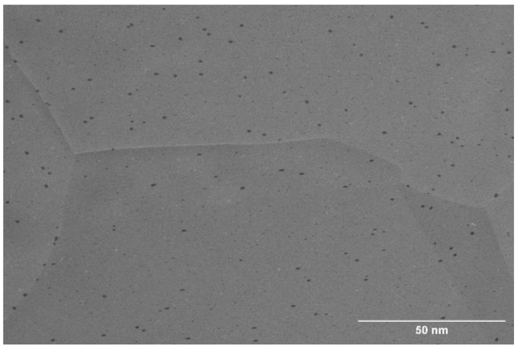

Figure S9. (a) Graphene placement inside Cu sheath, for CVD experiments. (b) SEM image of graphene regrowth, using Cu sheath (G_1E_5, 30 min)

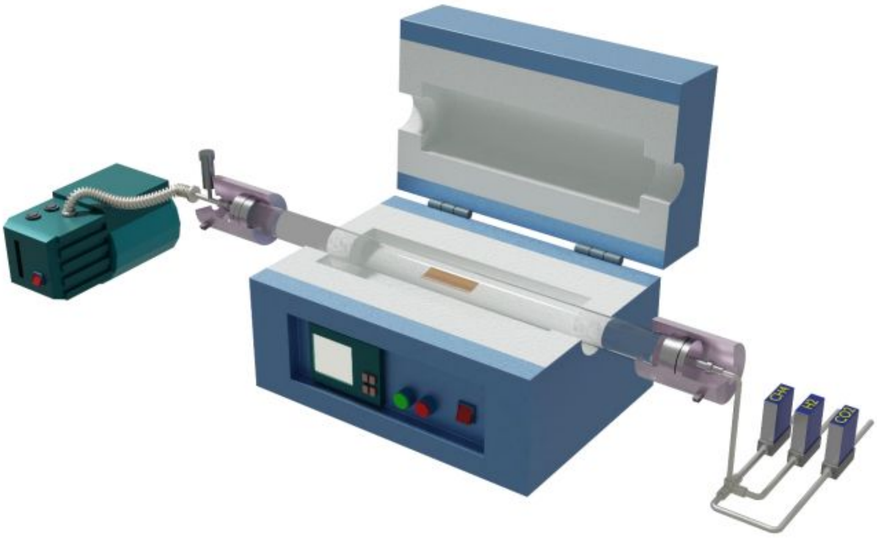

Figure S10. Schematic of CVD system

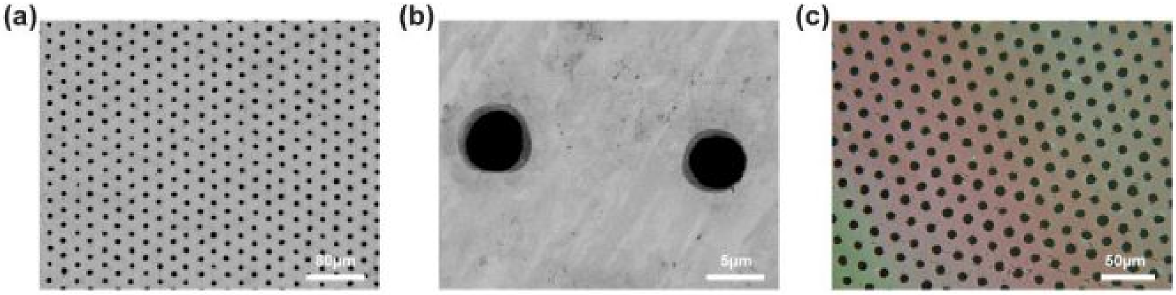

Figure S11. (a) Low and (b) high magnification SEM images of the polished porous W support. (c) Optical image of the PTMSP-supported graphene membrane on the W support.

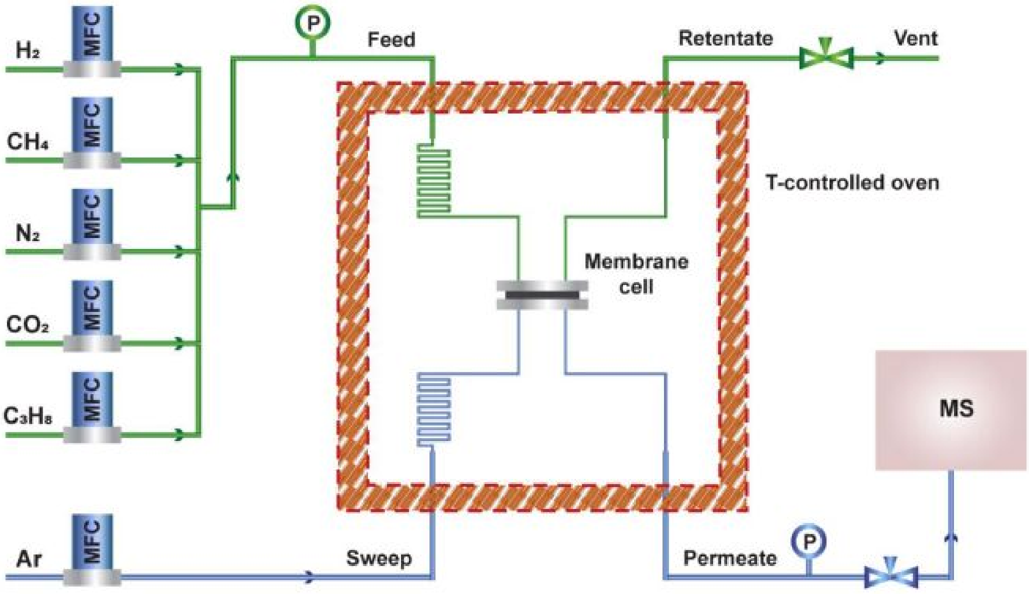

Figure S12. A schematic of the membrane testing setup.

Table S1. Comparison of H_2/C_3H_8 separation performance of porous graphene membrane.

Membrane Type	Note	H_2 permeance (GPU)	H_2/C_3H_8 selectivity (separation factor)	Reference
Porous Single Layer Graphene	*$G_1E_{0.5}$, 10 min*	8513	26	This work
Graphene	SLG	2580	207	[6]
Zeolite/MOF	ZIF-8	343	515	[7]
	ZIF-8	866	409	[8]
	MFI-Pt	776	7	[9]
	SAPO-34	687	27	[10]
Polymeric membranes	PIM-1	1000	8000	[11]
Mixed Matrix Membrane	20 wt% IZIF-8/PI	26.4	211.3	[12]
	20 wt% NZIF-8/PI	36.1	258.5	[12]

Table S2. Comparison of CO_2/N_2 separation performance of porous graphene membrane.

Membrane Type	Note	CO_2 permeance (GPU)	CO_2/N_2 selectivity (separation factor)	Reference
Porous Single Layer Graphene	*$G_1E_{0.5}$, 10 min (cm-scale)*	850	18	This work
	$G_1E_{0.5}$, 10 min, after post treatment (cm-scale)	2647	18.1	
Commercial membranes	(Gen 1) Polaris®	1000	50	13
	(Gen 2) Polaris®	2000	49	14
	Prism	161	37	15
Polymeric membranes	Pebax2533/PEG-b-PPFPA	3330	22	16
	PEG/NH2-MIL-53	3000	34	17
Facilitated transport membranes	Ionic liquid on graphene	4000	20	18
	Amine-incorporated polymer	1450	185	19

4. Additional Notes

Note S1: Carbon isotope labeling and Raman spectroscopy analysis

Carbon isotope labeling combined with Raman spectroscopy mapping is an effective method for studying the growth behavior of graphene by tracking the spatial distribution of methane sources ($^{12}CH_4$ and $^{13}CH_4$)[20,21]. The Raman frequency of a carbon isotope mixture can be estimated using the following formula[22]:

$$\omega = \omega_{12} \sqrt{}(m_{12} / (n_{12} \cdot m_{12} + n_{13} \cdot m_{13}))$$

Here, ω denotes the Raman frequency of the mixed isotopes, ω_{12} is the Raman frequency for pure ^{12}C, m_{12} and m_{13} are the atomic masses of ^{12}C and ^{13}C respectively, and n_{12} and n_{13} represent their atomic proportions.

Since Raman frequencies are inversely proportional to atomic mass, replacing ^{12}C with the heavier ^{13}C causes a spectral shift to lower frequencies. This shift follows a factor of $\sqrt{12/13}$, resulting in an approximate 100 cm^{-1} downshift in the 2D Raman peak for ^{13}C compared to ^{12}C.

5. References

1. Khan, M. H. *et al.* Hydrogen Sieving from Intrinsic Defects of Benzene-Derived Single-Layer Graphene. *Carbon* **153**, 458–466 (2019).
2. Kocaman, C. *et al.* Scalable room temperature incorporation of CO2-selective ångström-scale pores in graphene for carbon capture. *Nat. Commun.* **16**, 10380 (2025).
3. Suk, J. W. *et al.* Transfer of CVD-Grown Monolayer Graphene onto Arbitrary Substrates. *ACS Nano* **5**, 6916–6924 (2011).
4. Villalobos, L. F. *et al.* Polybenzimidazole Copolymer Derived Lacey Carbon Film for Graphene Transfer and Contamination Removal Strategies for Imaging Graphene Nanopores. *Carbon* **173**, 980–988 (2021).
5. Hsu, K. J. *et al.* Multipulsed Millisecond Ozone Gasification for Predictable Tuning of Nucleation and Nucleation-Decoupled Nanopore Expansion in Graphene for Carbon Capture. *ACS Nano* **15**, 13230–13239 (2021).
6. Zhao, J. Etching Gas-sieving Nanopores in Single-Layer Graphene with an Angstrom Precision for High-Performance Gas Mixture Separation. *Sci. Adv.* **5**, 1–10 (2019).
7. Kim, M. *et al.* ZIF-8 nanoplate/6FDA-DAM membrane for hydrogen extraction from propane dehydrogenation process. *J. Membr. Sci.* **685**, 121952 (2023).
8. Kim, M. *et al.* ZIF-8 nanoplate/6FDA-DAM membrane for hydrogen extraction from propane dehydrogenation process. *J. Membr. Sci.* **685**, 121952 (2023).

9. Kim, S.-J. *et al.* One-Step Synthesis of Zeolite Membranes Containing Catalytic Metal Nanoclusters. *ACS Appl. Mater. Interfaces* **8**, 24671–24681 (2016).
10. Kim, S.-J. *et al.* Thin Hydrogen-Selective SAPO-34 Zeolite Membranes for Enhanced Conversion and Selectivity in Propane Dehydrogenation Membrane Reactors. *Chem. Mater.* **28**, 4397–4402 (2016).
11. He, S. *et al.* Intermediate thermal manipulation of polymers of intrinsic microporous (PIMs) membranes for gas separations. *AIChE J.* **66**, e16543 (2020).
12. Kim, M. *et al.* ZIF-8 nanoplate/6FDA-DAM membrane for hydrogen extraction from propane dehydrogenation process. *J. Membr. Sci.* **685**, 121952 (2023).
13. Merkel, T. C., Lin, H., Wei, X. & Baker, R. Power plant post-combustion carbon dioxide capture: An opportunity for membranes. *J. Membr. Sci.* **359**, 126–139 (2010).
14. White, L. S., Amo, K. D., Wu, T. & Merkel, T. C. Extended field trials of Polaris sweep modules for carbon capture. *J. Membr. Sci.* **542**, 217–225 (2017).
15. Janusz-Cygan, A., Jaschik, J., Wojdyła, A. & Tańczyk, M. The Separative Performance of Modules with Polymeric Membranes for a Hybrid Adsorptive/Membrane Process of CO2 Capture from Flue Gas. *Membranes* **10**, 309 (2020).
16. Scofield, J. M. P. *et al.* Development of novel fluorinated additives for high performance CO2 separation thin-film composite membranes. *J. Membr. Sci.* **499**, 191–200 (2016).
17. Xie, K. *et al.* Continuous assembly of a polymer on a metal–organic framework (CAP on MOF): a 30 nm thick polymeric gas separation membrane. *Energy Environ. Sci.* **11**, 544–550 (2018).
18. Guo, W., Mahurin, S. M., Unocic, R. R., Luo, H. & Dai, S. Broadening the Gas Separation Utility of Monolayer Nanoporous Graphene Membranes by an Ionic Liquid Gating. *Nano Lett.* **20**, 7995–8000 (2020).
19. Chen, K. K., Salim, W., Han, Y., Wu, D. & Ho, W. S. W. Fabrication and scale-up of multi-leaf spiral-wound membrane modules for CO2 capture from flue gas. *J. Membr. Sci.* **595**, 117504 (2020).

20. Bernard, S., Whiteway, E., Yu, V., Austing, D. G. & Hilke, M. Probing the experimental phonon dispersion of graphene using 12 C and 13 C isotopes. *Phys. Rev. B* **86**, 085409 (2012).

21. Whiteway, E., Yang, W., Yu, V. & Hilke, M. Time evolution of the growth of single graphene crystals and high resolution isotope labeling. *Carbon* **111**, 173–181 (2017).

22. Fan, S., Liu, L. & Liu, M. Monitoring the growth of carbon nanotubes by carbon isotope labelling. *Nanotechnology* **14**, 1118–1123 (2003).
